# MIPPIE: the mouse integrated protein–protein interaction reference

**DOI:** 10.1093/database/baaa035

**Published:** 2020-06-04

**Authors:** Gregorio Alanis-Lobato, Jannik S Möllmann, Martin H Schaefer, Miguel A Andrade-Navarro

**Affiliations:** 1 Faculty of Biology, Johannes Gutenberg University, Biozentrum I, Hans-Dieter-Hüsch-Weg 15, 55128 Mainz, Germany; 2 Human Embryo and Stem Cell Laboratory, The Francis Crick Institute, 1 Midland Road, NW1 1AT London, UK; 3 Department of Experimental Oncology, European Institute of Oncology IRCCS, Via Adamello 16, 20139 Milan, Italy

**Keywords:** protein-protein interactions, *Mus musculus*, mouse, protein interaction network, database

## Abstract

Cells operate and react to environmental signals thanks to a complex network of protein–protein interactions (PPIs), the malfunction of which can severely disrupt cellular homeostasis. As a result, mapping and analyzing protein networks are key to advancing our understanding of biological processes and diseases. An invaluable part of these endeavors has been the house mouse (*Mus musculus*), the mammalian model organism par excellence, which has provided insights into human biology and disorders. The importance of investigating PPI networks in the context of mouse prompted us to develop the Mouse Integrated Protein–Protein Interaction rEference (MIPPIE). MIPPIE inherits a robust infrastructure from HIPPIE, its sister database of human PPIs, allowing for the assembly of reliable networks supported by different evidence sources and high-quality experimental techniques. MIPPIE networks can be further refined with tissue, directionality and effect information through a user-friendly web interface. Moreover, all MIPPIE data and meta-data can be accessed via a REST web service or downloaded as text files, thus facilitating the integration of mouse PPIs into follow-up bioinformatics pipelines.

## Introduction

The living cell is a crowded environment in which cellular constituents are rarely acting as isolated molecules (1). In consequence, it is not surprising that protein–protein interactions (PPIs) play a crucial role in most cellular operations (2) and that their perturbation may result in disease phenotypes (3). The importance of PPIs is reflected in the projects aimed at charting proteome-scale maps of the interactome (4, 5) and the number of repositories (6–8) and databases (9) that facilitate the access to these maps. Among the latter is the Human Integrated Protein–Protein Interaction rErefence (HIPPIE), which integrates experimentally validated human PPIs and calculates a confidence score for each one of them (10–12).

The idea of accompanying protein interactions with confidence scores is implemented in several PPI databases and aids in the construction of reliable and high-quality subnetworks in a variety of organisms (human, mouse and yeast among them). For example, the STRING database scores protein pairs based on the probability that they participate in the same pathway (13), iRefWeb uses an homology/evidence-based metric (14), InWeb_InBioMap focuses on PPI reproducibility (15) and IID offers an evidence-based filter (16). HIPPIE’s confidence score stands out from the rest because it is the only one that, to the best of our knowledge, considers not only the amount but also the quality of the experimental techniques employed to measure each interaction (10). In addition, HIPPIE incorporates experimentally determined gene-tissue associations for the construction of context-specific networks that can be visualized and enriched with directionality and effect inferences within a single user-friendly web interface (11, 17).

In this manuscript, we describe the extension of HIPPIE’s infrastructure and scoring system to the most important mammalian research model, the house mouse (*Mus musculus*), via the Mouse Integrated Protein–Protein Interaction rErefence (MIPPIE). Due to their physiological and genetic similarity to humans (18), short breeding times and ease of maintenance, mice have been critical to our understanding of human biology and disease (19, 20). As a result, we decided to develop a resource dedicated to PPIs in mouse that facilitates the study of high-quality, meaningful and tissue-specific mouse interactomes and the comparison with their human counterparts.

The following sections provide a detailed account of the technologies that power MIPPIE, the way in which it integrates, annotates and scores protein interaction data and the tools that it provides for network visualization, enrichment and analysis. We also present two use cases that showcase how MIPPIE can be used to infer signaling pathways and how MIPPIE and HIPPIE networks can be juxtaposed to study PPI conservation.

## Materials and Methods

### Back end and front end

We used MySQL v15.1 Distrib 10.1.37-MariaDB to store MIPPIE’s relational database. To facilitate access to these data, we developed a web tool that is compatible with the most popular browsers (Chrome ≥61, Firefox ≥55, Opera ≥47, Safari ≥11, Internet Explorer ≥11 and Edge ≥42). We implemented the tool’s front end with HTML, CSS and JavaScript and the communication with the database via PHP v7.0.30. MIPPIE can be accessed by any standard computer connected to the Internet and, since most queries and operations are solved on the server side, it does not have major memory requirements.

### P‌PI data

MIPPIE integrates experimentally validated mouse PPIs made available by molecular interaction providers through the PSICQUIC webservice (21). Specifically, we queried PSICQUIC with the Bioconductor package of the same name using the official symbols of all mouse protein coding genes reported in the Ensembl Genes 92 database. Then, we filtered the data to keep interactions annotated with the PSI-MI categories Association (MI:0914), Physical Association (MI:0915), Direct Interaction (MI:0407) and Colocalization (MI:0403) (22). Since PSICQUIC retrieves protein pairs with provider-specific IDs, we used the mygene Bioconductor package (23) to obtain the Entrez, symbol, MGI ID and UniProt accessions of each protein, which are all valid identifiers for querying MIPPIE. Interactions in which one or both proteins did not map to a valid Entrez ID were discarded, as well as duplicates and proteins without interaction partners. The final result is a table with the interacting proteins, the interaction type, the experimental technique used to measure the interaction and the studies where the interaction has been reported.

### Mouse protein interactions in other organisms

For each PPI in MIPPIE, we identified interologs, i.e. interactions between mouse homologs in other organisms (24), using data from the Interologous Interaction Database (16). This information is an important component of MIPPIE’s confidence score (see below).

### MIPPIE’s confidence score.

PPIs in MIPPIE are accompanied by a confidence score that indicates the amount and quality of the evidence supporting the interaction. MIPPIE’s confidence score is a weighted sum of three sub-scores:

$$ \mathrm{S}={\mathrm{w}}_{\mathrm{s}}{\mathrm{s}}_{\mathrm{s}}+{\mathrm{w}}_{\mathrm{o}}{\mathrm{s}}_{\mathrm{o}}+{\mathrm{w}}_{\mathrm{t}}{\mathrm{s}}_{\mathrm{t}} $$

with *w_s_ + w_o_ + w_t_ = 1*. Each sub-score *s_i_* is a saturating function of the form

$$ {s}_i(n)=\frac{2}{1+{e}^{-{\alpha}_in}}-1\in \left[0,1\right) $$

with *α_i_* controlling how quickly the function approaches its maximum value of 1 (10–12).

Depending on the sub-score, *n* acquires different meanings. When *i = s*, *n* is the number of studies in which the interaction was reported. When *i = o*, *n* is the number of species in which homologs of the interacting mouse proteins also interact. Finally, when *i = t*, *n* is the sum of the reliability scores of the different techniques used to measure the PPI. These reliability scores, which range between 0 and 10, were assigned to each experimental technique by experts in PPI detection (10). As a result, assays such as nuclear magnetic resonance or light scattering have reliabilities of 10, two-hybrid or co-immunoprecipitation methods of 5 and microscopy- or RNAi-based approaches of only 1.

Based on a study showing that PPIs supported by two or more pieces of evidence are more likely to be real (4), we set *α_s_ = 1* such that the corresponding sub-score saturates when *n = 3*. On the other hand, we set *α_o_ = 1.5* so that *s_o_* saturates when *n = 2*. Finally, we set *α_t_ = 0.5* to ensure that the sub-score approaches saturation if at least two PPI detection techniques with mid-level reliability were used to measure the interaction. Furthermore, the weights of the sub-scores were set to *w_s_ = 0.6*, *w_o_ = 0.1* and *w_t_ = 0.3*, reflecting the importance of the contribution of each one to the total confidence score. These are the same values used by HIPPIE and were selected based on a grid search aimed at finding the combination that best scored a subset of PPIs in an adapted cross-validation scheme (see (10) for details).

We note that it is possible to re-score the entire MIPPIE database or a subset of it using user-specified reliability scores, weights and/or saturation parameters by means of the scripts available on the tool’s website (more details below).

### Functional and tissue annotation of protein interactions

To allow for the construction of context-specific protein networks, we annotated each PPI in MIPPIE with Gene Ontology (GO) terms (25), Medical Subject Headings (MeSH) and tissue specificity information. For the GO annotations, we downloaded the generic GO Slim ontology file from http://www.geneontology.org/page/go-subset-guide and the gene-GO associations from http://www.informatics.jax.org/gotools/data/input/MGIgenes_by_GOid.txt. For MeSH, we downloaded the tree numbers for category C (Disease) from ftp://nlmpubs.nlm.nih.gov/online/mesh/MESH_FILES/meshtrees/ and used gene-MeSH annotations from the MeSH.db v1.10.0 and MeSH.Mmu.eg.db v1.10.0 Bioconductor databases. Finally, we employed gene expression data in 75 tissues and 35 cell types from the FANTOM5 promoter atlas (26).

We associated an interaction with a GO term or MeSH disease heading when both interactors are annotated with the given term/heading or with its children in the ontology hierarchy (17). A gene was considered to be present in a tissue if its expression was at least one TPM, a common threshold for gene expression calling (27, 28). If the products of two genes interact and are expressed in the same tissue, the PPI is assumed to take place in that tissue (17).

### Directionality and protein interaction effect

MIPPIE integrates directionality and interaction effect data from the SIGnaling Network Open Resource (SIGNOR) v2.0 (29). We extracted source-target relationships for PPIs only and whether the source activates or inhibits the target (up- or down-regulation effects, respectively). In addition, we labeled proteins in MIPPIE as receptors and/or transcription factors based on data from the Cell Surface Protein Atlas (30) and the Animal Transcription Factor Database (AnimalTFDB) v3.0 (31), respectively. These annotations are important for the pathway reconstruction tool offered by MIPPIE on its NETWORK QUERY tab (more details below).

### Network visualizations

We use Cytoscape.js (32), a JavaScript-based library, as a visualization component in MIPPIE. The results of a query can be represented as a network in which nodes are proteins and links between them are PPIs. Nodes are labeled with protein names and links with their MIPPIE confidence score. Users can interact with the network visualization (e.g. they can move nodes or adjust the zoom) and export it to commonly used graphics file formats (more details below).

## Results

### Database statistics

**Figure 1:**
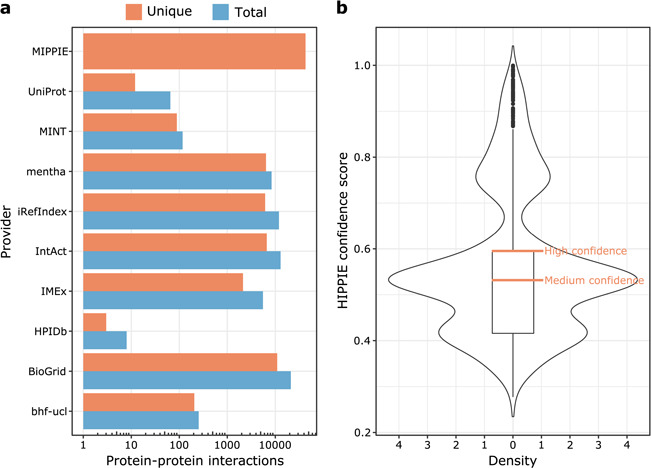
(**a**) MIPPIE stores 42 610 unique PPIs that originate from nine different molecular interaction providers. Some PPIs are reported by more than one provider, so the plot shows the total and unique number of interactions contributed by each one. (**b**) Distribution of confidence scores assigned to the 42 610 PPIs in MIPPIE. Interactions with scores above the median of the distribution are a mix of medium and high-quality PPIs, whereas interactions with scores above the upper quartile of the distribution are high quality PPIs only.

The current version of MIPPIE comprises 42 610 interactions between 10 886 proteins. These PPIs originate from nine different molecular interaction providers (see Figure 1a) and were integrated into MIPPIE via the PSICQUIC webservice (see Methods). The MIPPIE confidence score is not evenly spread among the 42 610 PPIs. To take this into account, we defined confidence levels based on quartiles of the score distribution (see Figure 1b). This means that the medium confidence threshold corresponds to the median (0.53) and the high confidence threshold to the upper quartile (0.6). In MIPPIE, it is possible to query for PPIs with one of these pre-defined confidence levels, as well as user-defined thresholds or no thresholds at all.

### Browsing MIPPIE

**Figure 2:**
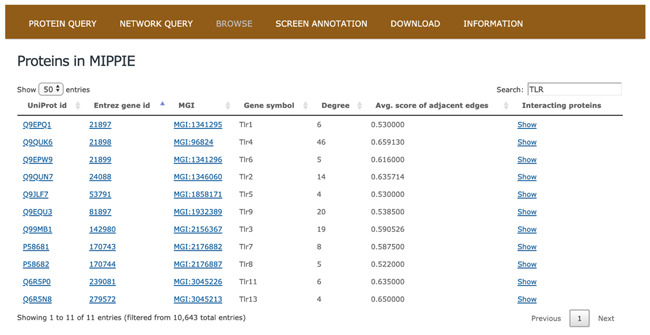
The BROWSE tab in MIPPIE shows a table with the proteome available in the database. Each protein in the table is listed with its UniProt, Entrez, MGI and Symbol identifiers, as well as its degree (number of interaction partners), the average MIPPIE score of the interactions in which the protein is involved and a link to explore the network around the protein.

The BROWSE tab in MIPPIE provides an overview of all the proteins with PPI data (see Figure 2). The MIPPIE proteome is shown as a table with one protein per row that can be filtered using the text box on the top-right corner. In addition, the table can be sorted by any of its columns. This tab helps to readily identify the most and least connected proteins in MIPPIE thanks to the Degree column. Also, it can be used to verify if there are any PPIs in MIPPIE for a protein of interest and explore them by clicking `Show’ on the last column.

### Protein queries

**Figure 3:**
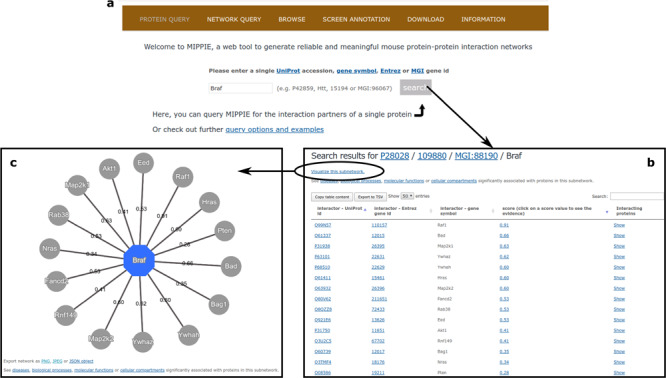
(**a**) On MIPPIE’s PROTEIN QUERY tab, users can enter a valid protein identifier to retrieve the interaction partners of that protein, if any. (**b**) MIPPIE generates a table with the query results and provides a link to generate a visual representation of the table (**c**).

MIPPIE welcomes its users with the PROTEIN QUERY tab (see Figure 3a). Once a user enters a valid protein identifier and clicks `search’, MIPPIE generates a table with the interaction partners of that protein, if there are any in the database. This table shows the different protein identifiers associated to these partners, the MIPPIE confidence score for each interaction and the option to retrieve the interactors of each partner (see Figure 3b). Clicking on the confidence score generates a report with details about the origin of the interaction: publications reporting it, experimental techniques used to measure it and presence in other organisms. The PROTEIN QUERY results page also provides a link to perform disease or GO enrichment analyses of the resulting network. For the former, MIPPIE resorts to the GS2D web tool (33) and for the latter to PANTHER (34). MIPPIE sends the relevant identifier of the query protein and its interactors to these tools and they perform a statistical test to check if they are over-represented in biomedical literature associated with a disease or in GO terms, respectively. Finally, it is possible to generate a graphic visualization of the result by clicking on `Visualize this subnetwork’ (see Figure 3c).

### Network queries

The NETWORK QUERY tab offers more advanced search and filtering options. On this tab, the user can take full advantage of MIPPIE’s protein and PPI annotations to construct condition-specific subnetworks.

**Figure 4:**
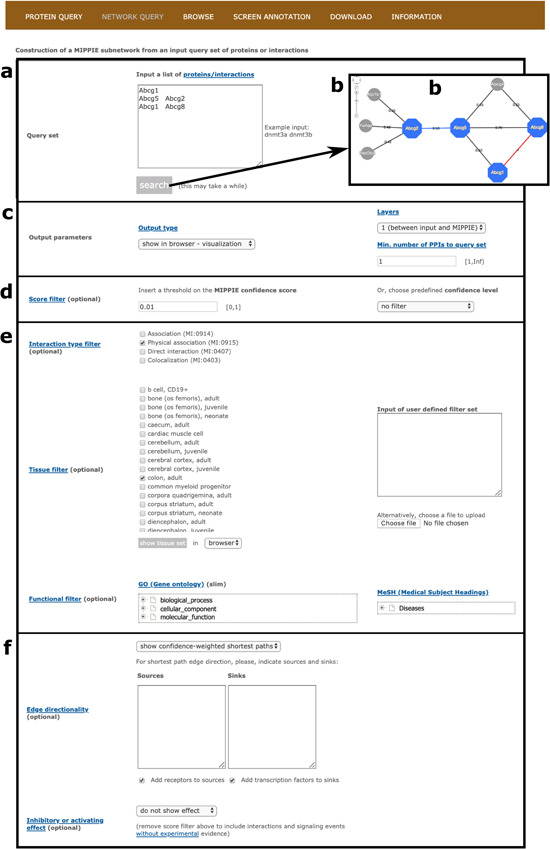
MIPPIE’s NETWORK QUERY tab offers the most advanced search and filtering options. (**a, b**) Users can type in or upload a list of proteins or protein pairs to quickly generate a visual representation of the network they form. (**c**) The results can be explored in a variety of output formats and constrained to 0-, 1-level neighbors or to a minimum number of interactions to the query set. (**d**) In addition, the quality of the output network can be fine-tuned with confidence score filters. (**e, f**) This tab also allows for the construction of tissue-, function- or disease-specific networks and for the prediction of interaction directionalities and/or effects.

First of all, the user must input a list of mouse proteins or protein pairs of interest or upload a file with such a list (see Figure 4a). Clicking `Search’ at this point will generate a network visualization of the interactions between the input proteins and their first-level neighbors based on default parameters (see Figure 4b). Note that for pairs of proteins in the input list, MIPPIE checks if there are reported interactions between them. If there are, they are highlighted in blue and accompanied by their MIPPIE confidence score. If there are not, they are highlighted in red with a pseudo-score of −1.

Second, it is possible to choose from different output formats (web-based table similar to the one obtained on the PROTEIN QUERY tab, tab-separated text file or tab-separated file in PSI-MI format), retrieve interactions between the input set only and consider first-level neighbors that interact with a minimum number of proteins from the input set (see Figure 4c). Also, PPIs with MIPPIE confidence score below a certain user-defined threshold can be discarded or a pre-defined threshold can be set instead (see Methods and Figure 4d). The choice of a threshold is application-dependent but if MIPPIE is being used in an area of biology in which false positives are unacceptable, we recommend the use of the `high confidence’ pre-defined cutoff. Conversely, users can construct networks without any pre-defined threshold and then, based on domain knowledge, apply more stringent filters to remove PPIs that are not relevant to the context of interest.

Third, interaction type, tissue and functional filters can be applied to construct tissue-, GO- or disease-specific networks based on protein and PPI annotations (see Methods). For example, one might be interested in physical PPIs between the products of nuclear genes that are expressed in the colon and have been associated with colorectal neoplasms (see Figure 4e).

Finally, MIPPIE offers an algorithm to infer edge directionality based on shortest paths between user defined sources and sinks or between proteins classified as receptors and transcriptions factors (see Methods). To infer edge directionality, all pairwise shortest paths between source and sink proteins in the resulting output network are calculated. If at least one shortest path passes through an edge of the network, it is considered to be directed with source-to-sink orientation. Edges with conflicting signs are not assigned directionality (17). Users can choose between two types of shortest path inference algorithms: `show unweighted shortest paths’ and `show confidence-weighted shortest paths’. In the former, all shortest paths with the same number of edges (not considering edge weights) are highlighted. In the latter, only the path with the highest cumulative confidence score is shown. Alternatively, known edge directions and PPI effects from the SIGNOR database can be overlaid on the constructed network (see Methods and Figure 4f). These annotations are reflected in the visualization output with different node colors and edge caps.

### Screen annotation

The SCREEN ANNOTATION tab represents a useful means to check if interactions detected in a screening have been already reported by others. The user must upload a tab-, comma- or semicolon-separated file of PPIs and MIPPIE will add an additional column indicating if the PPI is in the database (MIPPIE confidence score) or not (−1). The resulting file is automatically downloaded to the user’s computer.

### Downloading query results and data

Protein and network queries (if the selected output type is `show in browser – text’) can be copied or downloaded as tab-separated files by clicking on the corresponding buttons on the top left side of the results table (see Figure 3b). Network visualizations can be exported to the PNG, JPEG and JSON formats. Additionally, the DOWNLOAD tab provides links to download the entire MIPPIE database, the protein meta-data and annotations, as well as the scores assigned to the experimental techniques for measuring PPIs. Users can also re-score all the PPI data using their own parameters or perform more sophisticated queries by downloading the scripts available on the same tab.

### REST web services

To facilitate the integration of query results into more complex bioinformatics pipelines, users can access MIPPIE via its REST web service. REST requests must adhere to the following template:

http://cbdm-01.zdv.uni-mainz.de/~galanisl/mippie/queryMIPPIE.php?**proteins**=xxx,xxx;xxx|xxx&**layers**=xxx&**conf_thres** = xxx&**out_type** = xxx.

The parts of the template in boldface correspond to required parameters:

- proteins = one or more proteins of interest separated by `,’, `;’ or `|’ (mandatory)
- layers = 0 to query interactions within the input set or 1 to query interactions between the input set and MIPPIE (optional, default = 1)
- conf_thres = only protein interactions with confidence scores above this threshold, which ranges between 0 and 1, are considered (optional, default = 0)
- out_type = the query output format. *Browser* shows the list of interactions in a table in MIPPIE, *viz* shows a network visualization, *mitab* generates a MITAB file and *conc_file* generates a simple tab-separated text file (optional, default = *conc_file*)

### Example use cases

#### Inference of signal transduction pathways with MIPPIE.

This use case demonstrates the use of expression data and shortest path computations in MIPPIE’s NETWORK QUERY tab to infer a condition-specific signaling pathway. The list of proteins of interest is formed by Stk4, Lats2 and Tead4. These gene products are core members of the Hippo signaling cascade, whose activity is crucial for the first cell fate decisions in mouse early embryo development (35). When the Hippo pathway is active, the Stk4 kinase (also known as Mst1) in complex with Sav1 phosphorylates Lats2, which in turn phosphorylates Yap1 and Wwtr1 (also known as Taz) inhibiting their nuclear localization and driving the specification of pluripotent cells that will give rise to the embryo proper (36). In contrast, weak Hippo signaling allows nuclear accumulation of Yap1, which activates Tead4 and induces the specification of the trophectoderm cell lineage that will give rise to the placenta (36). MIPPIE does not include the trophectoderm in its tissue filter list but we can use an *in vitro* model of this cell type instead: `trophoblast stem cell line B1 differentiation’. Finally, we enable the directionality inference by unweighted shortest paths using Stk4 as source and Tead4 as target.

**Figure 5:**
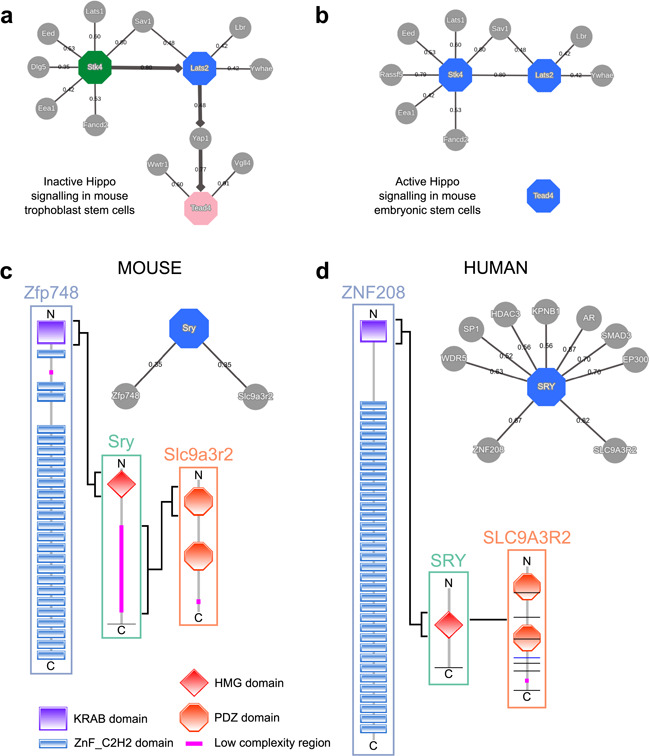
MIPPIE correctly reproduces the chain of Hippo signaling events that lead to (**a**) trophoblast specification or (**b**) pluripotency maintenance via the construction of cell type-specific networks with directionality inference using the NETWORK QUERY tab. (**c**) Interaction partners of the mouse Sry and (**d**) the human SRY proteins as reported in MIPPIE and HIPPIE, respectively. The accompanying diagrams highlight the domains that mediate conserved interactions.

The resulting network visualization correctly reproduces the chain of Hippo signaling events that lead to the trophectoderm specification (see Figure 5a). On the other hand, if we set the tissue filter to `stem cell’, an *in vitro* model of the pluripotent cell lineage, we obtain a network that resembles the active version of the pathway in which Yap1 is excluded from the nucleus and is unable to activate Tead4 (see Figure 5b).

#### Protein interaction comparison between human and mouse with HIPPIE and MIPPIE

To illustrate how MIPPIE and HIPPIE can help comparing interactions between organisms, we can use the Sry protein (sex determining region Y). This protein is one of the most different with respect to sequence conservation when comparing orthologs from mouse and human (37). While both orthologs have an HMG box DNA-binding domain at the N-terminal, the mouse ortholog has an additional low complexity region with Q-rich repeats, specific to and highly polymorphic in *Mus* species (see Figure 5c). This region is necessary for sex determination in the mouse (38).

Since polyQ regions and tandem repeats have a tendency to be involved in protein interactions (39, 40), one could expect that this low complexity C-terminal expansion in the murine Sry protein allows for different interactions compared to the shorter human Sry. The combined use of MIPPIE and HIPPIE can help us to address this question.

MIPPIE’s PROTEIN QUERY reports two partners for murine Sry: Slc9a3r2 and Zfp748. Clicking the confidence score of each PPI shows that each one is linked to one publication, which helps to understand their associated experimental evidence and functional implications. The interaction between Sry and Slc9a3r2 should highlight the sequence differences between Sry in mouse and human because the mouse-specific Q-rich region is reported to bind the PDZ1 domain of Slc9a3r2 (Figure 5c) (41). The other interactor, Zfp748, binds to the HGM box of Sry via a Krueppel-associated box (KRAB) N-terminal domain (Figure 5c) (42). This interaction could be expected to be replicated in human because the HGM domain is conserved.

HIPPIE’s PROTEIN QUERY reports nine interactors for human SRY. Surprisingly, human SRY also interacts with human SLC9A3R2 but using a different region (Figure 5d) (43). Regarding the interaction with Zfp748, no protein named Zfp748 is found in the list of nine interactors of human Sry, but one of them is ZNF208. This zinc-finger protein contains an N-terminal KRAB domain followed by tandem C2H2 domains and, like murine Zfp748, it interacts with SRY with its KRAB domain (Figure 5d) (44). Mouse Zfp748 and human ZNF208 are not orthologs, but a sequence similarity search of the human proteome using the mouse protein finds ZNF208 as best hit. Therefore, this indicates that the interaction is conserved between human and mouse.

## Conclusion

The active network of PPIs within a living cell is pivotal in determining how it reacts to environmental stimuli (2). With the aim to provide a platform devoted to the investigation of PPIs in mouse, we extended the concepts and infrastructure put forward by the well-established HIPPIE database to this model organism. In MIPPIE, users have access to 42 610 interactions between 10 886 proteins. Each one of these PPIs has been assigned a score that summarizes the number of studies that support the occurrence of the interaction, the number of other species in which the interaction also takes place and the quality of the experimental technique used to measure the interaction. This last component of the confidence score, together with the tools featured in the above use cases (construction of context-specific networks, directionality inference and comparison with human interactomes via HIPPIE), is one of the key characteristics that differentiates MIPPIE from other PPI resources.

Updates of all components of the MIPPIE database (PPIs, annotations and scores) are going to be performed biannually. However, all previous versions of the resource will be archived and accessible through the DOWNLOAD tab. Finally, we are planning to provide more integration between MIPPIE and HIPPIE in order to facilitate network comparative analyses.

### Availability

MIPPIE is freely available at https://cbdm.uni-mainz.de/mippie.

## Author contributions

M.H.S. designed and implemented the HIPPIE database and its web-based interface. G.A.L. adapted HIPPIE’s infrastructure to MIPPIE’s needs and was also in charge of downloading and processing all the necessary data and populating the MIPPIE database. J.S.M. annotated the PPIs with disease, effect and directionality information. G.A.L., J.S.M. and M.A.N. were in charge of the use cases presented in the manuscript. G.A.L., M.H.S. and M.A.N. wrote the manuscript. G.A.L. and M.A.N. supervised the project. M.A.N. provided the resources for the realization of the project. All authors read and approved the final version of the manuscript.
